# Diagnostic brain-to-liver [^18^f]fdg uptake ratio predicts survival in multiple myeloma: A retrospective study

**DOI:** 10.1007/s00259-026-07844-z

**Published:** 2026-03-21

**Authors:** Maria Emilia S. Takahashi, Tiago P. dos Santos, Christopher Cralcev, Eliana Miranda, Marcos Paulo D. S. Silva, Felipe C. Souza, José Barreto C. Carvalheira, Carmino A. de Souza, Celso Dario Ramos

**Affiliations:** 1 Instituto de Física Gleb Wataghin, Universidade Estadual de Campinas (UNICAMP), Campinas, SP Brazil; 2 Faculdade de Ciências Médicas, Universidade Estadual de Campinas (UNICAMP), Campinas, SP Brazil; 3 Centro de Hematologia e Hemoterapia, Universidade Estadual de Campinas (UNICAMP), Campinas, SP Brazil; 4 Departamento de Radiologia e Oncologia, Universidade Estadual de Campinas (UNICAMP), PO Box 6149, Zeferino Vaz Avenue, S/N, 13080-000 Campinas, Brazil; 5 Serviço de Medicina Nuclear, Universidade Estadual de Campinas (UNICAMP), Campinas, SP Brazil

**Keywords:** Multiple myeloma, [^18^F]FDG PET/CT, Brain-to-liver ratio, SUV

## Abstract

**Purpose:**

[¹⁸F]FDG PET/CT has been widely used in oncology for diagnosis and treatment monitoring. Reduced cerebral [¹⁸F]FDG uptake has been observed in patients with disseminated malignancies, potentially associated with the Warburg effect and elevated lactate levels. This study investigated the prognostic relevance of the brain-to-liver [¹⁸F]FDG uptake ratio (BLR) in MM patients who underwent PET/CT at diagnosis.

**Methods:**

BLR was calculated as the ratio between the mean standardized uptake value (SUV) of the whole brain and that of the liver. A total of 72 patients were retrospectively included in the study (58% male; median age 65 years (IQR55;70); 67% were classified as ISS stage III).

**Results:**

BLR, as a continuous variable, showed a statistically significant difference between groups according to sex (*p* = 0.004), overweight status (*p* = 0.005), and ISS stage (*p* = 0.03), and was negatively correlated with β₂-microglobulin (*r*= − 0.42, *p* < 0.001). With a median follow-up of 23 months (IQR 8; 65), 74% patients had died from MM-related causes. Patients with BLR > 2.7 demonstrated superior 60-month overall survival (52%vs.10%, *p* = 0.002) and progression-free survival (19%vs.3%, *p* = 0.003), respectively. Multivariate Cox regression confirmed BLR (HR 1.86 95%CI: 1.03–3.33, *p* = 0.038 (OS); HR 1.93 95%CI: 1.13–3.29, *p* = 0.016 (PFS))as an independent factor and the use of autologous hematopoietic cell transplantation (HCT) as consolidation for both OS and PFS. In addition, ISS stage III was also a prognostic factor for OS.

**Conclusion:**

Higher brain-to-liver [¹⁸F]FDG uptake ratio (> 2.7) at diagnosis predicts better clinical outcomes in multiple myeloma. BLR is significantly associated with established clinical markers of tumor burden in multiple myeloma. These findings suggest that BLR is a feasible and reproducible metric with potential prognostic value in multiple myeloma.

**Supplementary Information:**

The online version contains supplementary material available at 10.1007/s00259-026-07844-z.

## Introduction

Positron emission tomography combined with computed tomography using fluorine-18 fluorodeoxyglucose ([¹⁸F]FDG PET/CT) has been widely applied in oncology for diagnosis, disease staging, and treatment monitoring [1]. As [¹⁸F]FDG is a glucose analogue, this imaging modality enables the evaluation of the functional and metabolic status of tumors, reflecting their glycolytic activity [2, 3]. Recent studies in lymphoma imaging have highlighted the relevance of cerebral FDG uptake as a surrogate marker of systemic metabolic and inflammatory activity. Associations between brain FDG uptake, disease burden, and treatment response suggest that alterations in cerebral metabolism may carry prognostic information [4, 5]. These observations provide a conceptual basis for exploring ratios between brain and reference-organ uptake, such as the liver, as potential imaging biomarkers in other hematologic malignancies.

In multiple myeloma (MM), [¹⁸F]FDG PET/CT is routinely used for staging and response assessment. Alongside magnetic resonance imaging, it is one of the preferred imaging techniques for detecting and evaluating bone lesions in this hematologic malignancy [6–8].

In [¹⁸F]FDG PET studies, the liver is commonly employed as an internal reference standard for visual classification of lesion uptake across the body due to its stable and reproducible metabolic activity over time [9, 10]. This property makes it a reliable intra-patient reference for comparisons based on the standardized uptake value (SUV). The SUV represents the concentration of a radiotracer in a given voxel normalized to the injected activity and patient weight, with all parameters corrected to a common reference time [11].

Beyond tumor evaluation, several studies have reported reduced cerebral [¹⁸F]FDG uptake in patients with disseminated malignancies [12–16]. This phenomenon has been attributed to systemic metabolic alterations, including the diversion of radiotracer from the brain to hypermetabolic tumor tissues, as well as mechanisms related to the Warburg effect and elevated lactate levels [17, 18].

This study aimed to evaluate the association between the brain-to-liver [¹⁸F]FDG uptake ratio (BLR) at diagnosis and clinical and prognostic variables in patients with multiple myeloma.

## Patients and methods

### Study design and population

This is a retrospective study conducted with patients diagnosed with multiple myeloma who underwent whole-body [^18^F]FDG PET/CT imaging at diagnosis at the Hospital of the Universidade Estadual de Campinas (UNICAMP) between June 2013 and July 2018. The study included patients aged 18 or older who had not received prior treatment or were just starting therapy for the disease. The exclusion criteria were images with backup errors and images presenting acquisition artifacts (e.g., head movement).

The study was approved by the local Ethics Committee (CAAE 97966618.5.0000.5404) and conducted in accordance with the Declaration of Helsinki.

### Image acquisition protocol

Patients fasted for approximately 6 h before image acquisition. An intravenous injection of 0.12 mCi/kg of [^18^F]FDG was administered, and whole-body scans were performed 60 min post-injection using a Biograph mCT40 PET/CT scanner (Siemens Medical, USA). PET images were acquired for 1.5 min per bed position and reconstructed using a standard iterative algorithm (3D-OSEM with PSF and TOF modeling; 2 iterations, 21 subsets). The corresponding CT images acquired in the same session were used for attenuation correction.

### Brain-to-liver ratio measurement

BLR was calculated by dividing the mean brain SUV by the mean liver SUV for each patient on the [^18^F]FDG PET images (Eq. 1).

1 $$BLR=\frac{{meanSUV}_{brain}}{{meanSUV}_{liver}}$$

**Fig. 1:**
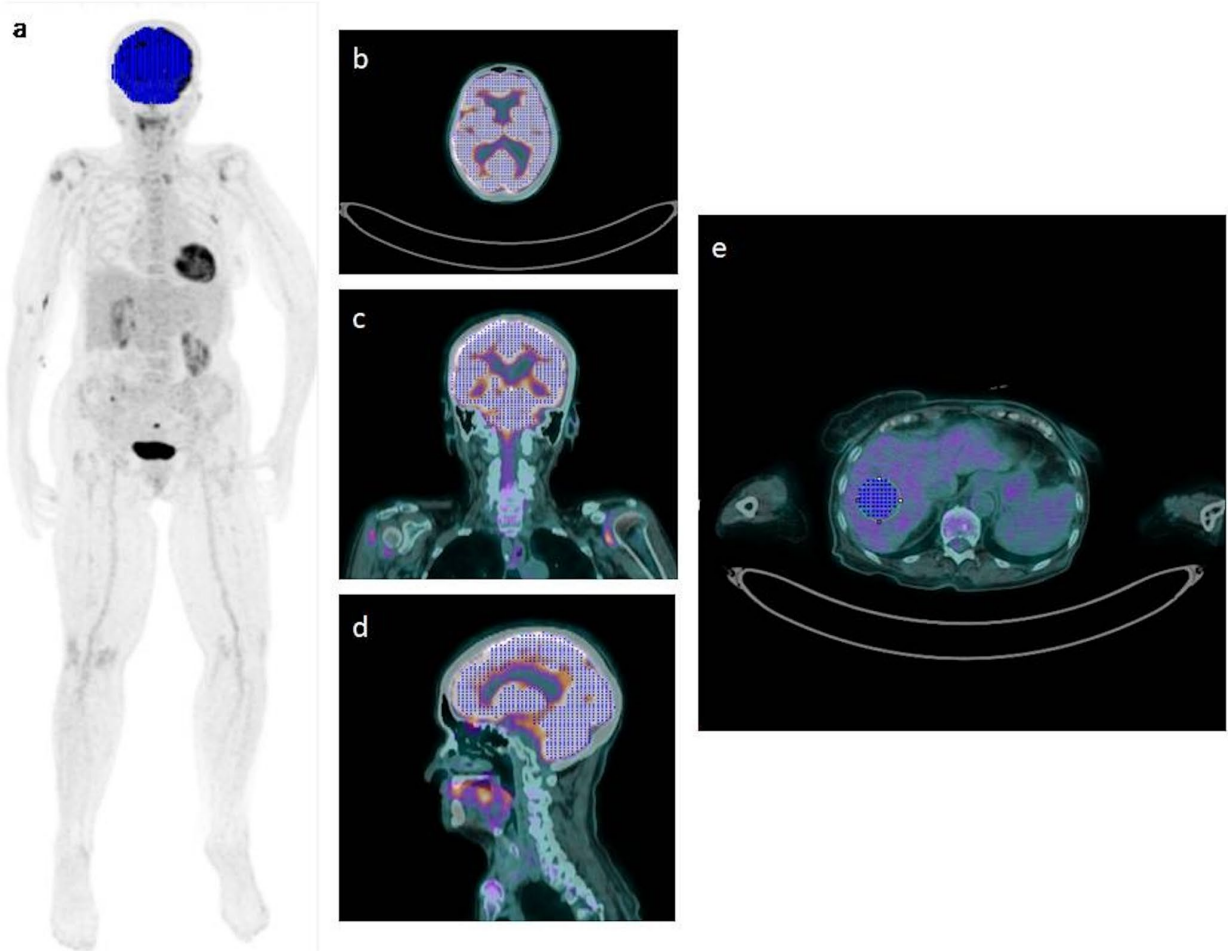
Image (a) shows the Maximum Intensity Projection (MIP) of the [¹⁸F]FDG PET scan, where the blue area represents the auto-segmented brain region used to calculate the mean SUV of this organ. The same segmented region is shown in the axial (b)**,** coronal (c), and sagittal (d) planes. Image (e) shows the fixed spherical volume, in blue, used to measure the mean hepatic SUV in the right lobe

The mean liver SUV was measured in a spherical region with a fixed volume of 34.3 mL placed in the right hepatic lobe, using the Beth Israel Plugin for FIJI [19, 20]. The mean brain SUV was determined through automatic segmentation using the Grow Mask method in the same software (Fig. 1). SUV measurements were derived from a brain segmentation predominantly representing gray matter rather than the entire brain.

### Statistical analysis

The patient cohort was characterized using descriptive statistics of demographic, clinical, and image-derived variables. Correlations between BLR and quantitative clinical variables were assessed applying Spearman’s rank correlation test. Differences between patient groups were analyzed using the Mann–Whitney and Kruskal-Wallis test with Dunn´s post-hoc comparisons, as appropriate. Kaplan–Meier estimators were used to calculate overall survival (OS) and progression-free survival (PFS), where OS was defined as the interval from the date of diagnosis to the last follow-up or death, and PFS as the interval from the date of diagnosis to clinical progression, death or last follow-up. Survival curves were compared using the log-rank test. Statistical significance was defined as a p-value ≤ 0.05. All analyses were conducted using RStudio (version 2025.09.2). The follow-up data were updated in January 2025.

## Results

### Study population

**Fig. 2:**
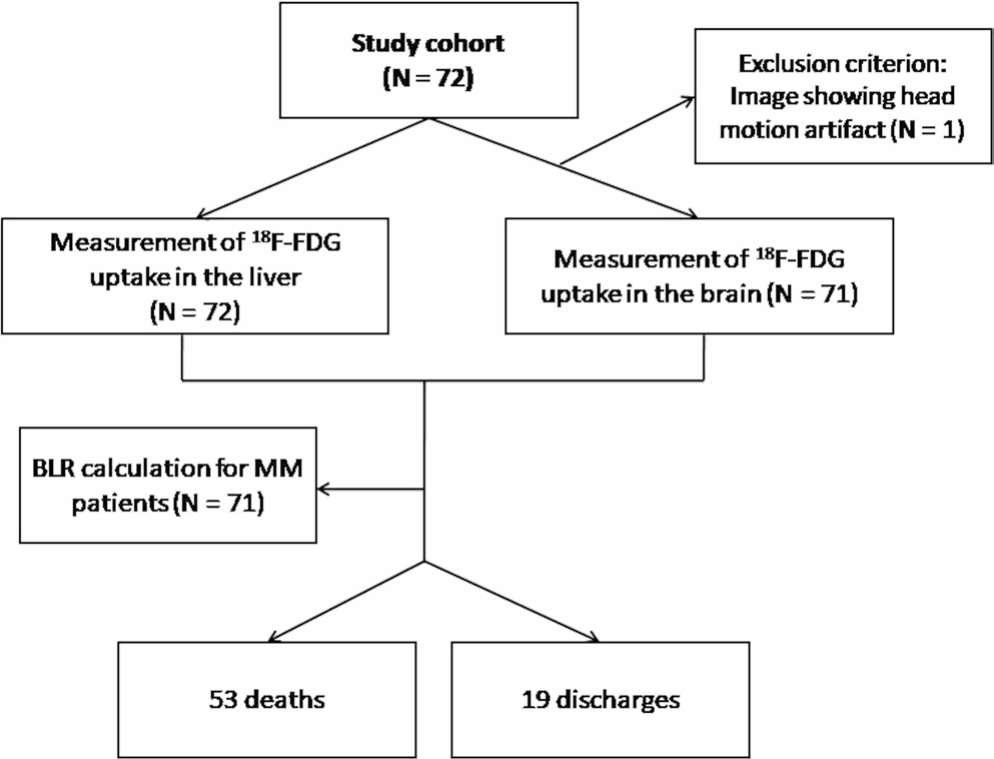
Study flowchart. BLR: brain-to-liver ratio; MM: multiple myeloma

A total of 72 patients with multiple myeloma were included in the study (Fig. 2); 42 (58%) were male, and the median age at the time of the PET/CT scan was 65 years (IQR:55;70). At diagnosis, 67% of patients were classified as ISS stage III. Additional baseline characteristics of the study population are presented in Table 1 and treatment characteristics and response data are summarized in Table 2.

**Table 1:** Clinical and demographic characteristics of patients with multiple myeloma atdiagnosis

Variables	n= 72 (%)
Sex, male	42 (58)
Race/ethnicity*: white	51 (71)
Age, years**	65 (55;70)
BMI, kg/m²**	24.3 (22;27)
BMI kg/m²	(n= 69)
<18.5	2 (3)
18.5–24.9	34 (47)
25.0–29.9	20 (31)
>30.0	13 (19)
Hemoglobin,g/dl**	9.9 (7.9;11.2)
Creatinine, mg/d**	1.1(0.78;2.67)
Creatinine clearance, mL/min **	63.8(39.2;86.7)
Calcium, mg/dL, **	9.4 (8.9;10.0)
β2-microglobulin, mg/dL **	0.8 (0.4;1.2)
LDH, U/l **	193 (135;360)
Albumin, g/dL **	3.4 (2.9;3.9)
CRP, mg/dL **	1.4 (0.41;5.8)
NLR **	1.9 (1.16;3.96)
MLR **	0.2 (0.16;0.31)
PLR **	139.6 (81.4;182.5)
Plasma cells, % **	29.5 (14.0;52.7)
Bone lesions	63 (88)
Type of M protein	
IgG/Kappa	32 (45)
IgG/Lambda	6 (8)
Only light chain/ Kappa	12 (17)
Only light chain/ Lambda	6 (8)
IgA/Kappa	6 (8)
IgA/Lambda	10 (14)
ISS	
I	11 (15)
II	13 (18)
III	48 (67)
ECOG	(n=71)
0	34 (48)
1	25 (35)
2	9 (13)
3	3 (4)

*Self-declared (White, Black or Multiracial, Asia or Indigenous)

** Median(IQR:interquartile range)

*BMI* body mass index, *CRP* C-reactive protein, *ECOG* eastern cooperative oncology group performance status, *ISS* international staging system, *LDH* lactate dehydrogenase, *MLR* monocyte-to-lymphocyte ratio,* NLR* neutrophil-to-lymphocyte ratio, *NR* not reported, *PLR* platelet-to-lymphocyte ratio, *SD* standard deviation

**Table 2:** Features of treatment and responses of multiple myeloma patients

Variables	*n* = 72
Number of chemotherapy cycles*(*n* = 70)	6 (6;8)
Chemotherapy Type	*n* = 70
CTD	54 (75)
MPT	6 (8)
VTD	3 (4)
VD	3 (4)
CY + PDS	2 (3)
CDT + VTD	2 (3)
Bortezomib – 1st line	8 (11)
Response after 1st line	*n* = 70
CR or VGPR	19 (28)
PR	33 (49)
MR or SD	10 (13)
PD	08 (10)
Autologous HCT	19 (26)
Maintenance (*n* = 68)	15 (22)

* Median (IQR: Interquartile range), *CTD* Cyclophosphamide+Thalidomide+Dexamethasone, *MPT *Melphalan+Prednisolone+ Thalidomide, *VTD* Bortezomib+Thalidomide+Dexamethasone, *VD *Bortezomib+Dexamethasone, *CY + PDS *Cyclophosphamide + Prednisolone, *CR* Complete remission, *VGPR* very good partial remission, *PR* Partial remission, *MR* minimum response, *SD* stable disease, *PD* progression disease, *HCT* hematopoietic cell transplantation,

Autologous hematopoietic cell transplantation (HCT) after first-line therapy was performed in 26% of the cohort. The reasons for the low rate of HCT were as follows: age over 75 years (29%), death (23%), disease progression (15%), high comorbidity index (13%), patient refusal (10%), loss to follow-up (8%), and mobilization failure (2%).

### Brain and liver [^18^F]FDG uptake and their ratio

**Fig. 3:**
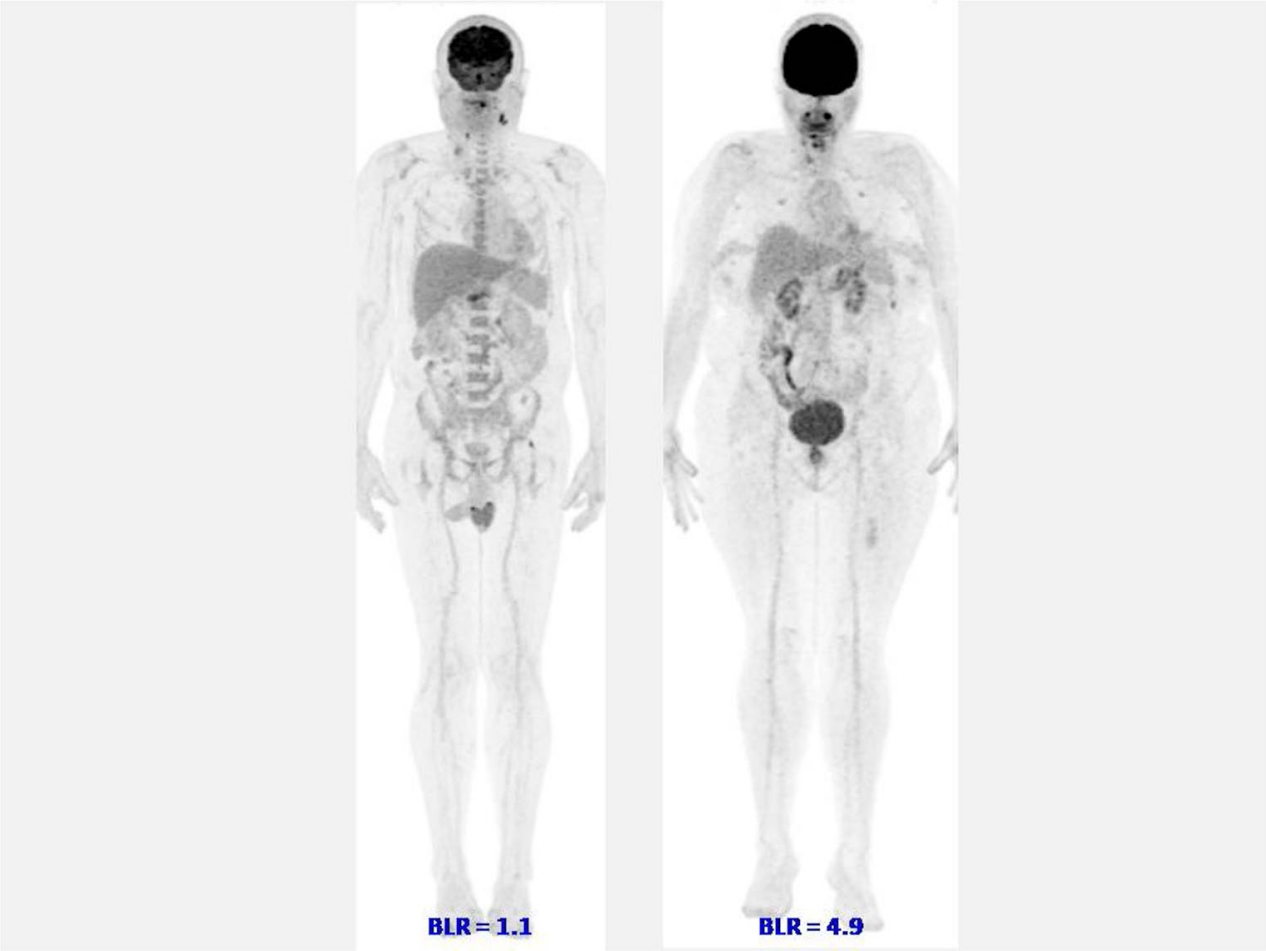
Left: Patient with a low BLR value (1.1); male, 47 years old at diagnosis; ISS stage III; light chain/lambda;β₂-microglobulin = 2.80 mg/dL; PFS and OS = 7.7 months. Right: Patient with a high BLR value (4.9); female, 52 years old at diagnosis; ISS stage III; IgG/kappa; β₂-microglobulin = 1.13 mg/dL; PFS = 93.1 months and OS = 113.4 months

The mean SUV values obtained for the liver and brain, as well as the calculated BLR values are presented in Table 1S (supplemental). The lowest BLR value found was 1.05 whereas the highest value obtained was 6.45 (IQR:2.15;3.36). The Maximum Intensity Projection (MIP) images from the [¹⁸F]FDG PET/CT scans of patients with low and high BLR values are presented in Fig. 3.

One patient was excluded from the brain SUV calculations and, consequently, from the BLR calculation due to the presence of a motion artifact on the ¹⁸F-FDG PET/CT image, which rendered the image quality insufficient for variable extraction.

### Association between BLR and clinical and demographic variables

Quantitative clinical and demographic variables were correlated with BLR using Spearman’s correlation test. Correlations were considered statistically significant for p-values ≤ 0.05. Weak correlations, that is, those with a Spearman’s coefficient magnitude below 0.4 were observed for BMI, creatinine, creatinine clearance, C-reactive protein (CRP), and percentage of plasma cells. A moderate *negative* correlation was observed between β₂-microglobulin and BLR (*r*=–0.42, *p* < 0.0001) (Table 2S - supplemental).

Categorical clinical variables were analyzed in relation to BLR using the Mann–Whitney or Kruskal–Wallis tests, with statistical significance set at *p* < 0.05. BLR was significantly associated with sex, overweight status and international stage system (ISS) [21]. On average, female patients showed higher BLR values compared to males. Patients with overweight also presented higher BLR compared to those without this condition. Furthermore, BLR was significantly associated with the staging (ISS), and in Dunn’s post-hoc test, patients in the ISS III group had significantly lower BLR values compared to those in the ISS I group (Table 3S - supplemental).

### Survival outcomes

**Fig. 4:**
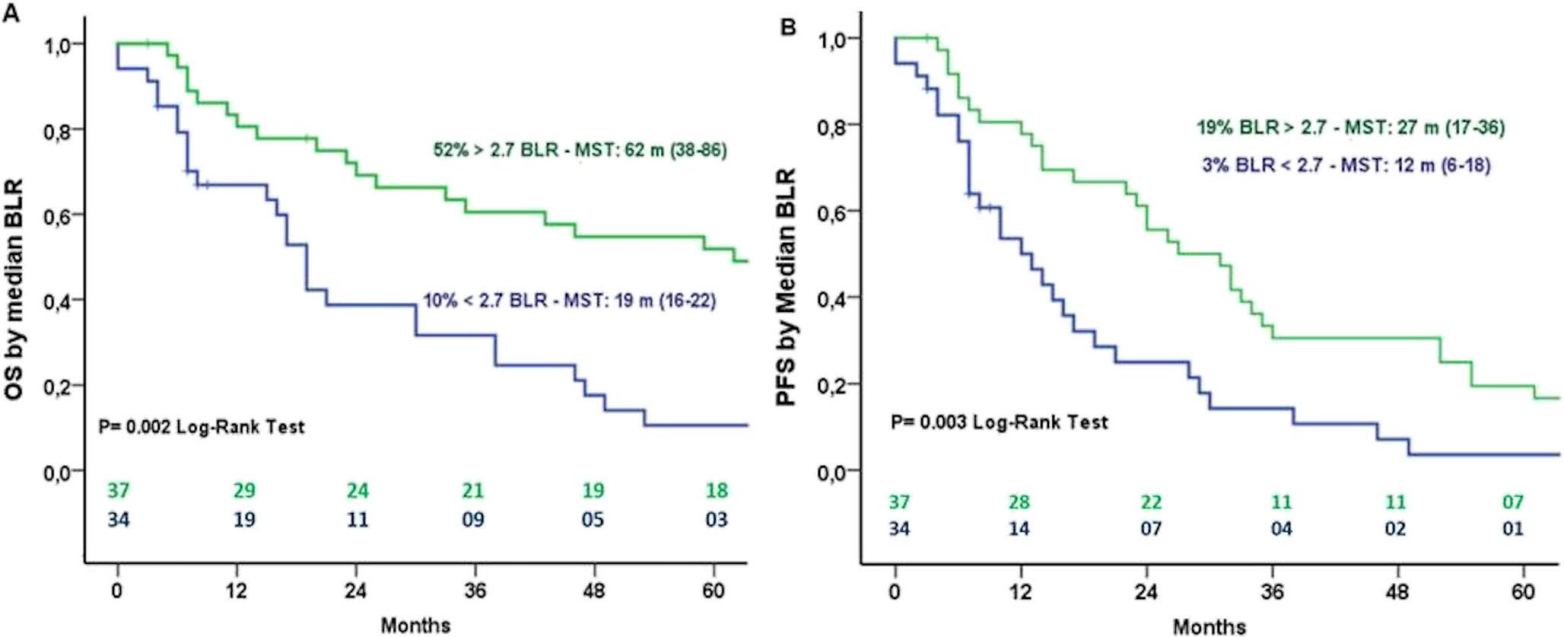
A. Overall Survival by median BLR; B**.** Progression-Free Survival by median BLR; MST: median survival time

Kaplan–Meier analysis stratified by the median BLR value (2.7) revealed a significant association between BLR and 60-month OS and PFS. Patients with BLR values higher than 2.7 had better outcomes:52% vs. 10% (*p* = 0.002) for OS and 19% vs. 3% (*p* = 0.003) for PFS, respectively. Median OS was 62 months in patients with higher BLR compared with 19 months in those with lower BLR (Fig. 4A), and median PFS was 27 months vs.12 months, respectively (Fig. 4B).

In the multivariate Cox regression analysis of factors associated with OS, lower BLR values were associated with a higher risk of death (HR = 1.86, 95%CI: 1.03–3.33, *p* = 0.038), stage 3 – ISS (HR = 2.02, 95%CI: 1.05–3.86, *p* = 0.033) and HCT as consolidation (HR = 3.39, 95%CI: 1.61–7.12, *p* = 0.001) presented in Table 3.

**Table 3:** Uni and Multivariate Cox Regression analysis of factors associated with overall survival (death)

Variable	*n*	HR	95% CI	*p*	HR	95% CI	*p*
		Univariate			Multivariate*		
Median BLR < 2.7	37	2.40	(1.36–4.25)	0.003	1.86	(1.03–3.33)	0.038
Sex: male	42	1.75	(0.99–3.06)	0.050			
BMI	70	(-) 0.91	(0.86–0.97)	0.006			
ISS		(-) 0.85	(0.74–0.98)	0.024			
I	11	reference					
II	13	1.48	(0.69–3.14)	0.31			
III	48	2.40	(1.27–4.52)	0.007	2.02	(1.05–3.86)	0.033
Nº CT cycles	72	(-) 0.82	(0.71–0.95)	0.010			
HCT as consolidation	19	0.25	(0.12–0.53)	< 0.0001	0.29	(014-0.62)	0.001
Type of M protein	72						
IgG	38	2.39	(1.38–4.15)	0.002			
IgA	16	2.09	(1.14–3.82)	0.017			
Light chain	18	reference					

*BLR* brain-to-liver ratio, *BMI *Body mass index, *CI* confidence interval, *CT *chemotherapy, *HCT* hematopoietic cell transplantation, *HR* hazard ratio, *ISS* international stage system

* Adjusted model by sex, age at diagnosis, *ECOG *(eastern cooperative oncology group), and *ISS *score

For PFS, BLR was also identified as an independent factor associated with progression and mortality (PFS), lower BLR values (HR = 1.93, 95%CI: 1.13–3.29, *p* = 0.016) and HCT as consolidation (HR = 2.55, 95%CI: 1.39–4.68, *p* = 0.002) shown in Table 4.

**Table 4:** Uni and Multivariate Cox regression analysis of factors associated with progression-free survival (progression and death)

Variable	*n*	HR	95% CI	*p*	HR	95% CI	*p*
		Univariate			Multivariate*		
Median BLR < 2.7	37	2.19	(1.29–3.71)	0.004	1.93	(1.13–3.29)	0.016
Sex: male	42	1.68	(1.01–2.81)	0.045			
BMI	70	(-) 0.93	(0.88–0.98)	0.007			
ISS		(-) 0.85	(0.74–0.98)	0.024			
I	11	reference					
II	13	1.28	(0.66–2.46)	0.46			
III	48	1.56	(0.92–2.67)	0.098			
Nº CT cycles	72	(-) 0.87	(0.77–0.98)	0.022			
HCT as consolidation	19	0.35	(0.19–0.64)	0.001	0.39	(0.21–0.71)	0.002
Type of M protein	72						
IgG	38	2.58	(1.53–4.35)	< 0.0001			
IgA	16	2.38	(1.32–4.27)	0.004			
Light Chain	18	reference					

*BLR* brain-to-liver ratio, *BMI* body mass index, *CI *confidence interval, *CT* chemotherapy, *HCT* hematopoietic cell transplantation, *HR *hazard ratio, *ISS *international stage system

* Adjusted model by sex, age at diagnosis, *ECOG* (eastern cooperative oncology group) and *ISS* score

## Discussion

This study included patients with newly diagnosed MM between 2013 and 2018 who were treated at a reference center within the Brazilian public health system. In our cohort, most patients (75%) received the CVD regimen (cyclophosphamide, thalidomide, and dexamethasone) as first-line therapy. Only a small proportion (11%) had access to the proteasome inhibitor (PI) bortezomib, an important drug class for this disease. Although bortezomib was approved by the FDA in 2008 for first-line treatment of MM, it became approved and available in the Brazilian public health system only in 2020.

The overall response rate (complete response, very good partial response, and partial response) was 77%, a reasonable outcome considering the therapeutic options available at that time. For comparison, Morgan et al. [22] reported overall response rates with the CTD regimen of 82.5% before HCT and 91.6% after auto-HCT.

After auto-HCT, 22% of patients received maintenance therapy with thalidomide for 1 to 2 years, until disease progression or treatment intolerance. Although not commonly used when lenalidomide is available, thalidomide remains a valuable option, as demonstrated by Maiolino et al. [23] who showed improvements in progression-free survival.

The high proportion of ISS stage III patients in this cohort reflects elevated β₂-microglobulin levels and indicates an advanced disease profile with substantial tumor burden at diagnosis. This enrichment in high-risk disease likely influenced both baseline metabolic findings and clinical outcomes. In this context, autologous HCT remains a cornerstone of therapy, as it has consistently demonstrated the ability to reduce disease progression and improve survival, particularly when performed after effective induction therapy. Notably, despite only 26% of patients undergoing HCT in our cohort, this intervention emerged as an independent protective factor in multivariate analysis, in line with previous reports [21, 24, 25].

BLR is a relatively simple parameter to implement, since the tools required for measuring SUV are already available in most nuclear medicine image analysis software. Moreover, BLR calculation does not require data pre-processing or complex mathematical procedures. This metric has already been reported by other authors in different clinical contexts. Previous studies have shown that a reduction in cerebral [¹⁸F]FDG uptake may occur in patients with extensive or disseminated malignancies, such as malignant lymphoma and poorly differentiated adenocarcinoma [12–14, 26].

The decision to use a fixed VOI for liver segmentation, rather than automatic segmentation as applied to the brain, was based on EANM recommendations [27] and on the study by de Souza et al. [28], which showed that whole-liver segmentation—using Hounsfield unit thresholds or artificial intelligence—does not significantly affect mean liver SUV and may introduce artifacts and increase processing time. In contrast, no standardized definition exists for brain SUV as a reference tissue, supporting the use of an automatically segmented brain VOI predominantly including gray matter.

In multiple myeloma, a recent study by Dingli et al. [29] demonstrated a poorer response to CAR-T cell therapy in patients who, at any time during treatment, presented a BLR below 2.5. Although the threshold value of 2.5 proposed by the authors was not directly derived from the statistical dispersion of BLR, it is very close to that observed in our study.

Dingli et al. [29] hypothesized that the reduced cerebral [¹⁸F]FDG uptake could be associated with the Warburg effect, in which the tumor’s high glycolytic activity competes for the available glucose, thereby reducing its systemic availability for cerebral metabolism.

Another possible explanation is that more aggressive cases of multiple myeloma may be associated with increased neoplastic lactate production, a consequence of the aerobic glycolysis characteristic of the Warburg effect [30].

Lactate is capable of crossing the blood–brain barrier and can be utilized by the brain as an energy substrate, concomitantly with glucose. Thus, the reduction in cerebral [¹⁸F]FDG uptake observed on PET/CT may reflect preferential lactate utilization as an alternative energy source rather than a true decrease in cerebral metabolic activity [16, 31, 32]. Interestingly, no correlation was observed between BLR and LDH, the enzyme responsible for converting pyruvate to lactate. This lack of association may indicate that increased lactate levels in some patients result from alterations in production or clearance rather than from significant tissue injury, of which LDH is a marker [33].

Another well-known factor that can reduce [¹⁸F]FDG uptake on PET/CT is hyperglycemia [34]. However, this factor was not considered in our cohort, since blood glucose levels are routinely measured in all patients undergoing [¹⁸F]FDG PET/CT at the Hospital of the Universidade Estadual de Campinas. Patients with glucose levels above 180 mg/dL are rescheduled for a new scan.

Another possible explanation for the decreased cerebral [¹⁸F]FDG uptake and increased hepatic uptake could be metabolic syndrome (MS), as demonstrated by Nam et al. [35]. Such cases could result in lower BLR values. However, our data showed an opposite pattern to that described in MS: the BLR was correlated with body mass index (− 32%, *p* = 0.008). These findings do not support the hypothesis that low BLR values are associated with the presence of MS in our cohort.

In addition, BLR showed significant associations with key biological and physiological parameters, including β₂M levels (− 42%, *p* < 0.0001), a well-established tumor burden marker in multiple myeloma [36] with higher β₂M levels being associated with lower BLR. Significant inverse associations were also observed with C-reactive protein (CRP; −38%, *p* = 0.014) and serum creatinine (− 33%, *p* = 0.004), as well as a significant association with sex (*p* = 0.01). Although these findings indicate robust statistical relationships, they remain observational in nature, and further studies are required to determine whether these associations are causal or merely correlative.

In the multivariable regression analysis, low BLR (< 2.7) remained independently associated with worse overall and progression-free survival, together with advanced disease stage (ISS III) for overall survival, while HCT as consolidation was associated with improved outcomes. These findings suggest that BLR provides prognostic information beyond established clinical risk factors, although confirmation in larger cohorts is warranted.

Taken together, these observations indicate that variations in BLR are more likely attributable to the biological behavior of the disease rather than to systemic factors unrelated to multiple myeloma that could secondarily influence cerebral metabolism.

The retrospective design of this study imposes some inherent limitations, such as the inability to systematically measure serum lactate levels at diagnosis. In addition, the absence of longitudinal monitoring of BLR over time limits the assessment of its dynamic prognostic value. Finally, the lack of minimal residual disease (MRD) evaluation may represent a potential confounding factor in survival analyses and could influence the interpretation of long-term outcomes.

Another considerable limitation is the use of methods to determine an optimal cutoff for BLR; attempts using the Youden index and the ROC curve were unsuccessful, most likely due to the size of our sample. However, the multivariate analysis results indicating BLR values as an independent factor influencing OS and PFS encourages us to prospectively consider the use of this factor. Although both BLR and HCT were independently associated with outcomes, exploratory analyses did not identify a statistically significant interaction between these variables; however, the study was underpowered to reliably detect such effects.

These findings indicate that BLR measurement is a promising, easily implementable, and noninvasive parameter that warrants further investigation in other oncologic contexts and in prospective studies to better elucidate its clinical relevance.

In conclusion, the brain-to-liver [^18^F]FDG uptake ratio (BLR) proved to be a simple and reproducible metric, showing significant correlations with clinical markers of tumor burden. Higher BLR values were associated with improved overall and progression-free survival, suggesting that this parameter may indirectly reflect tumor activity.

## Supplementary Information

Below is the link to the electronic supplementary material.

Supplementary Material 1

## Data Availability

The datasets used and analysed during the current study are available from the corresponding author on reasonable request.
