# Supplementary material for: Diagnostic brain-to-liver [18f]fdg uptake ratio predicts survival in multiple myeloma: A retrospective study
Source: Eur J Nucl Med Mol Imaging. 2026 Mar 21;53(8):5033–41. doi: 10.1007/s00259-026-07844-z (PMC13249654; doi:10.1007/s00259-026-07844-z)
Supplement: Supplementary file 1 — Supplementary Material 1 [file 259_2026_7844_MOESM1_ESM.docx]

**Table 1S**. Descriptive statistics of BLR and the mean SUV values obtained for the liver and brain.

| **Variable** | **N** | **Mean±SD** | **Median [IQR / min-max]** |
| --- | --- | --- | --- |
| Liver SUVmean | 72 | 2.3 ± 0.5 | 2.27 [1.98;2.52 / 1.23-4.37] |
| Brain SUVmean | 71 | 6.3 ± 2.0 | 5.99 [4.57;7.96 / 3.62-11.6] |
| Brain-to-liver Ratio (BLR) | 71 | 2.8 ± 0.9 | 2.72 [2.15;3.36 / 1.05-6.45] |

**Abbreviations:** BLR: Brain-to-liver Ratio; SUV: standardized uptake value, SD: standard deviation; IQR: interquartile range.

**Table 2S**. Results of Spearman’s correlation test between BLR and quantitative variables.

| **Variable** | **Spearman Correlation Coefficients** | **p** |
| --- | --- | --- |
| Age | 0.02 | 0.84 |
| BMI | **-0.32** | **0.008** |
| Hemoglobin | 0.17 | 0.15 |
| Creatinine | **-0.33** | **0.004** |
| Creatinine clearance | **0.30** | **0.010** |
| Calcium | -0.009 | 0.94 |
| β2-microglobulin | **-0.42** | **<0.0001** |
| LDH | -0.02 | 0.85 |
| Albumin | 0.06 | 0.58 |
| CRP | **-0.38** | **0.014** |
| NLR | -0.09 | 0.41 |
| MLR | -0.05 | 0.63 |
| PLR | 0.12 | 0.29 |
| % of plasma cells | **-0.23** | 0.05 |

**Abbreviations:** BMI: body mass index; CRP: C-reactive protein; LDH: lactate dehydrogenase, MLR: monocyte-to-lymphocyte ratio; NLR: neutrophil-to-lymphocyte ratio; PLR: platelet-to-lymphocyte ratio.

**Table 3S.** Statistical association between BLR and categorical variables.

| **Variables** | |  | **BLR** | | |
| --- | --- | --- | --- | --- | --- |
|  |  | **N** | **Mean±SD** | **Median [IQR]** | **p** |
| **Sex** | Male | 42 | 2.6±0.9 | 2.38 [2.02;3.14] | **0.017^a^** |
|  | Female | 28 | 3.1±0.8 | 3.26 [2.51;3.73] |  |
| **Race/ethnicity** | White | 49 | 2.9±0.9 | 2.78 [2.21;3.63] | 0.29^a^ |
|  | Non-White | 21 | 2.6±0.9 | 2.49 [1.96-3.17] |  |
| **Bone Lesions** | Yes | 60 | 2.8±0.9 | 2.74 [2.19;3.33] | 0.98^a^ |
|  | No | 08 | 2.8±1.1 | 2.81 [1.85;3.96] |  |
| **Overweight**  (BMI>25 kg/m²) | Yes | 32 | 3.1±1.0 | 3.17 [2.41;3.73] | **0.008^a^** |
|  | No | 36 | 2.6±0.8 | 2.39 [1.98;3.18] |  |
| **ISS** | I* | 11 | 3.4 ±0.5 | 3.65 [3.17;3.79] | 0.01^b^ |
|  | II | 13 | 2.7±0.9 | 2.74 [1.93;3.42] |  |
|  | III* | 46 | 2.7±1.0 | 2.44 [2.07;3.22] |  |
| **Type of M protein** | IgG | 38 | 2.9±0.7 | 2.96 [2.29;3;51] | 0.12^b^ |
|  | IgA | 14 | 2.5±0.8 | 2.26 [2.03;3.08] |  |
|  | Light chain | 18 | 2.8±1.2 | 2.56 [2.00;3.66] |  |

^a^ Mann-Whitney test; ^b^ Kruskal-Wallis’s test

*Dunn’s post-hoc test: BLR values in the ISS III group were significantly lower than those in the ISS I group

**Abbreviations:** BLR: brain-to-liver ratio; SD: standard deviation; IQR: interquartile range; ISS: International Staging System.
